# Intervention Strategies for Improving Patient Adherence to Follow-Up in the Era of Mobile Information Technology: A Systematic Review and Meta-Analysis

**DOI:** 10.1371/journal.pone.0104266

**Published:** 2014-08-06

**Authors:** Haotian Lin, Xiaohang Wu

**Affiliations:** State Key Laboratory of Ophthalmology, Zhongshan Ophthalmic Center, Sun Yat-sen University, Guangzhou, China; Swiss Tropical & Public Health Institute, Switzerland

## Abstract

**Background:**

Patient adherence to follow-up plays a key role in the medical surveillance of chronic diseases and affects the implementation of clinical research by influencing cost and validity. We previously reported a randomized controlled trial (RCT) on short message service (SMS) reminders, which significantly improved follow-up adherence in pediatric cataract treatment.

**Methods:**

RCTs published in English that reported the impact of SMS or telephone reminders on increasing or decreasing the follow-up rate (FUR) were selected from Medline, EMBASE, PubMed, and the Cochrane Library through February 2014. The impacts of SMS and telephone reminders on the FUR of patients were systematically evaluated by meta-analysis and bias was assessed.

**Results:**

We identified 13 RCTs reporting on 3276 patients with and 3402 patients without SMS reminders and 8 RCTs reporting on 2666 patients with and 3439 patients without telephone reminders. For the SMS reminders, the majority of the studies (>50%) were at low risk of bias, considering adequate sequence generation, allocation concealment, blinding, evaluation of incomplete outcome data, and lack of selective reporting. For the studies on the telephone reminders, only the evaluation of incomplete outcome data accounted for more than 50% of studies being at low risk of bias. The pooled odds ratio (OR) for the improvement of follow-up adherence in the SMS group compared with the control group was 1.76 (95% CI [1.37, 2.26]; P<0.01), and the pooled OR for the improvement of follow-up adherence in the telephone group compared with the control group was 2.09 (95% CI [1.85, 2.36]; P<0.01); both sets showed no evidence of publication bias.

**Conclusions:**

SMS and telephone reminders could both significantly improve the FUR. Telephone reminders were more effective but had a higher risk of bias than SMS reminders.

## Introduction

Follow-up refers to the timely surveillance of health status and guidance in a medication regimen by various methods among patients who visited or were visited by medical staff. [1] Adherence to follow-up (AFU) is most commonly measured as the follow-up rate (FUR), which is also called the attendance rate, [2] retesting rate, [3] or screen rate, [4] with different definitions and calculations according to the specific research background. As a medical process characterized by long-term observation, AFU plays an irreplaceable role in chronic disease management. [5]–[8] In addition to the treatment effect, AFU seriously affects clinical research implementation: participants who are enrolled but do not complete a trial (study attrition) can undermine the internal and external validities of the findings and cause bias when participants are not lost randomly but rather have certain characteristics. Loss to follow-up usually necessitates that more participants be enrolled to attain adequate power for the trial results to be valid, which may increase the trial’s cost or duration or delay important results [9].

Given the significance of AFU, studies were performed to investigate the measurements of and related factors influencing FUR [10] and, in particular, to explore effective, novel interventions to improve FUR in the era of mobile information technology. [11] This technology has greatly affected the way people live and work and has also been convenient for medical practice. [12] However, most published studies have focused more on adherence to medication and less on AFU, and few have simultaneously assessed the nature and relative effectiveness of compliance interventions across the broad spectrum of patient conditions and compliance measures. [13] In our previous randomized controlled trial (RCT; ClinicalTrials.gov, NCT01417819), we demonstrated a successful and practical intervention with short message service (SMS) reminders to significantly improve the AFU of families with clinically meaningful pediatric eye care in a setting with limited resources. [2] In the present study, we aimed to systematically evaluate the published RCTs reporting on the impact of AFU in patients with SMS and/or telephone reminders, both of which are the most used features, although varying in cost and convenience, in the era of mobile information technology.

## Methods

### Literature Sources

A comprehensive search of databases, including Medline, EMBASE, PubMed, and the Cochrane Library, was conducted using the search terms “telephone”, “text message”, “SMS”, “adherence”, “compliance”, “follow up”, and “attendance”. We also searched conference abstracts and the reference lists of the studies identified by the search. The latest search date was February 4, 2014. Two authors independently screened titles and abstracts to determine potential eligibility for this meta-analysis. When screening discrepancies occurred, consensus was achieved after further discussion.

### Inclusion and Exclusion Criteria

We carefully reviewed all potentially relevant articles, and inclusion was restricted to RCTs. The included studies described the impact of SMS or telephone reminders on increasing or decreasing the FUR, and the control group did not receive a reminder of any type. To avoid including duplicate data, the newest and most informative article was selected when multiple studies were conducted by the same authors.

### Date Extraction and Outcome Measure

Two authors independently extracted the data from the selected articles. The primary outcome was the FUR (also known as the attendance rate, retesting rate, nonattendance rate, or screen rate), defined as the proportion of patients attending their appointment at the originally scheduled time. [14] If the FUR was calculated more than once, according to different follow-up periods, the initial data were used. We abstracted or calculated the odds ratio (OR) in the intervention group compared with the OR in the controls as the primary effect measure for the study.

### Quality Assessment and Statistical Analysis

To determine whether the selected studies were appropriate for inclusion in the meta-analysis, two authors assessed each trial independently and resolved disagreements via consensus. The risk of bias in each trial was assessed according to Cochrane methodology, [15] considering random sequence generation, allocation concealment, the blinding of patients and personnel, incomplete outcome data, selective reporting, and other biases. The heterogeneity of each trial was determined through a visual inspection of forest plots and with a standard Chi^2^ test and an inconsistency (I^2^) statistic. [16] P values<0.05 indicated significant heterogeneity. Additionally, for I^2^<25%, we used fixed-effects meta-analysis to estimate the common OR (95% CI); for I^2^ = 25 to 75%, we used random-effects meta-analysis; and for I^2^>75%, because the heterogeneity was too great for a summary estimate to be calculated, subgroup analysis was needed. The statistical analysis was performed using the Rev Man Computer program (Version 5.0. The Cochrane Collaboration, 2008, The Nordic Cochrane Centre, Copenhagen, Denmark) using two-sided hypothesis testing with alpha = 0.5. For the dichotomous data, ORs were used.

## Results

**Figure 1:**
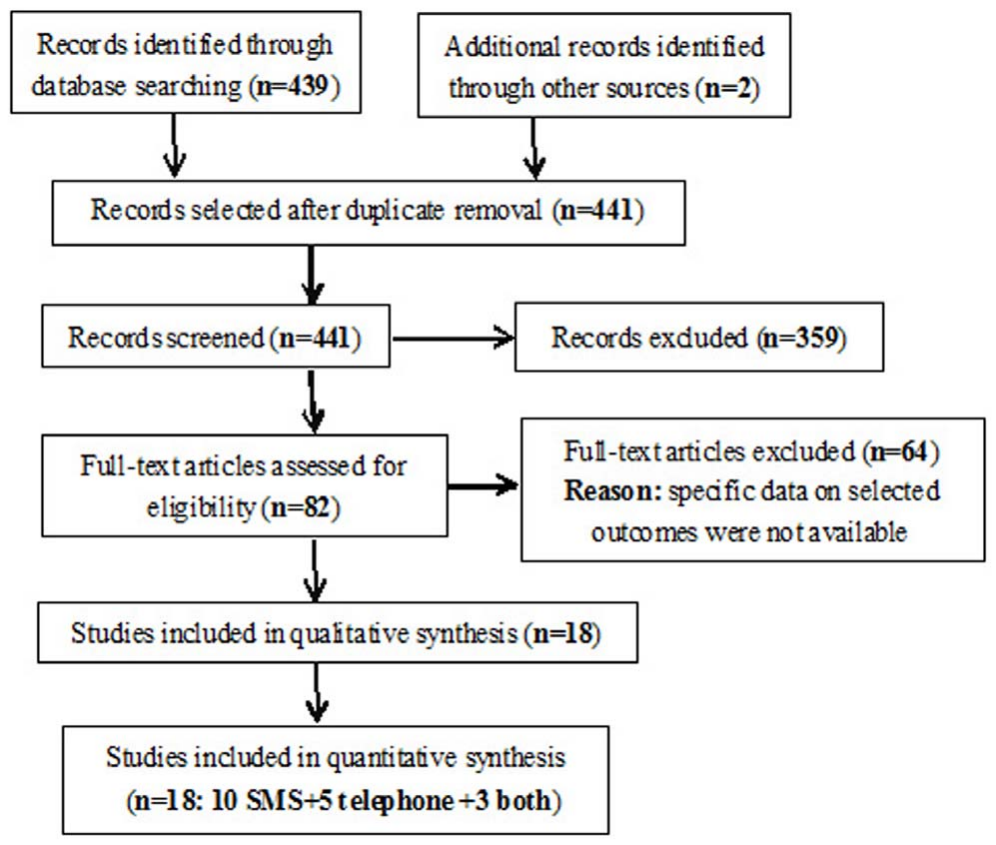
Flowchart of the included and excluded studies.

Of the 441 titles and abstracts screened, only 18 RCTs were identified in our systematic review, including 10 studies only focused on SMS reminders, 5 only focused on telephone reminders, and 3 focused on both SMS and telephone reminders (Figure 1). All 18 RCTs were published in English between 1995 and 2014 and were from 9 countries. The 13 RCTs [2], [3], [17]–[27] on SMS reminders included 3276 patients with and 3402 patients without SMS reminders, and the 8 RCTs [25]–[32] on telephone reminders reported on 2666 patients with and 3439 patients without telephone reminders. The most used (7/18) measurement index of adherence to follow-up was the attendance rate in the included studies, and the nonattendance rate and the attendance rate at the first appointment/visit were the second and third most used (Table 1).

**Table 1:** Main characteristics of the eligible studies included in the systematic review.

First author	Year	Country	Recruitmentperiod	Studydesign	Inclusion-exclusioncriteria	Consecutivepatients	Electronicreminder type	Measurement ofadherence to follow-up
Clough [23]	2014	Australia	N/A	RCT	Yes	Yes	SMS	Attendance rate at firstappointment
Downing [3]	2014	Australia	N/A	RCT	Yes	Yes	SMS	Retesting rate
Wang [24]	2014	China	Dec.2011–Mar.2012	RCT	Yes	N/A	SMS	Attendance rate
Narring [22]	2013	Switzerland	Nov.2010–Apr.2011	RCT	Yes	N/A	SMS	Proportion of unexplainedmissed appointmentswithout prior notification
Lin [2]	2012	China	Dec.2010–Aug.2011	RCT	Yes	Yes	SMS	Attendance rate at first visit
Odeny [19]	2012	America	Sep.2010–Apr.2011	RCT	Yes	N/A	SMS	Return rate at day 7
Prasad [20]	2012	India	Sep.2010–Dec.2010	RCT	Yes	N/A	SMS	Attendance rate
Taylor [21]	2012	Australia	N/A	RCT	Yes	N/A	SMS	Nonattendance rate
Liew [18]	2009	Malaysia	N/A	RCT	Yes	N/A	SMS	Nonattendance rate
Fairhurst [17]	2008	UK	Aug.2004–Feb.2005	RCT	Yes	N/A	SMS	Nonattendance rate
Goelen [32]	2010	Belgium	N/A	RCT	Yes	N/A	Telephone	Mammography rate
Roberts [31]	2007	UK	N/A	RCT	N/A	N/A	Telephone	Attendance rate
Sawyer [30]	2002	Australia	Aug.1998–Jan.1999	RCT	N/A	N/A	Telephone	Attendance rate
Vivier [29]	2000	America	Jul.1998–Sep.1998	RCT	Yes	N/A	Telephone	Proportion ofchildren immunized
Ferson [28]	1995	Australia	N/A	RCT	N/A	N/A	Telephone	Immunization rate
Chen [27]	2007	China	Apr.2007–May.2007	RCT	Yes	Yes	SMS+Telephone	Attendance rate
Leong [26]	2006	Malaysia	Apr.2005–Oct.2005	RCT	Yes	N/A	SMS+Telephone	Attendance rate
Bos [25]	2005	Netherlands	N/A	RCT	N/A	N/A	SMS+Telephone	Attendance rate

**Figure 2:**
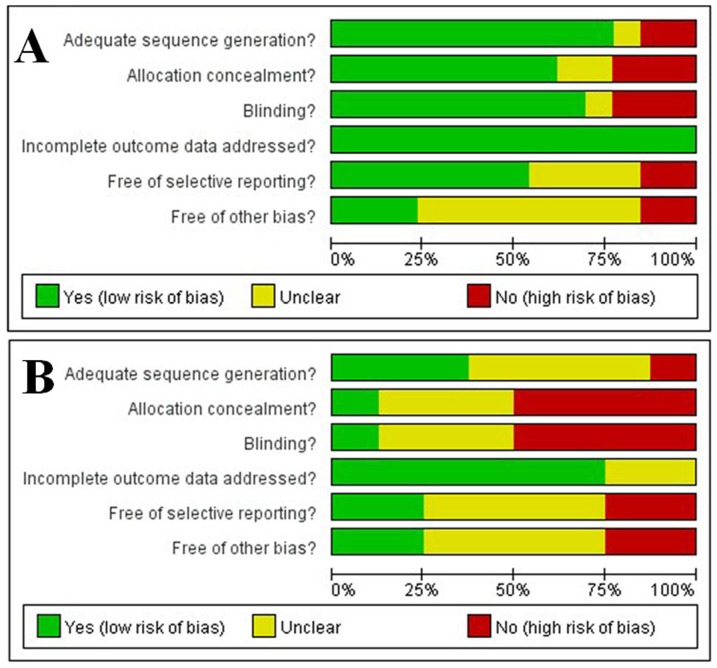
Risk-of-bias graphs. Panel A, evaluation of the study quality of RCTs on SMS reminders; Panel B, evaluation of the study quality of RCTs on telephone reminders. The green bar means reported and a low risk of bias, the yellow bar means unreported and a moderate risk of bias, and the red bar means unreported and a high risk of bias.

According to the Cochrane methodology, the risk of bias of the included studies was assessed by considering adequate sequence generation, allocation concealment, blinding, the evaluation of incomplete outcome data, lack of selective reporting, and lack of other biases (Figure 2). For SMS reminders for the respective Cochrane factors, the studies at low risk of bias had values (a quantitative index of the risk of bias, range 0–100%) of 76.9%, 61.5%, 69.2%, 100%, 53.8%, and 23.1%; the studies with unreported features and a moderate risk of bias had values of 7.7%, 15.4%, 7.7%, 0%, 30.8%, and 61.5%; and the studies at high risk of bias had values of 15.4%, 23.1%, 23.1%, 0%, 15.4%, and 15.4%. For telephone reminders for the respective Cochrane factors, the studies at low risk of bias had values of 37.5%, 12.5%, 12.5%, 62.5%, 25.0%, and 25.0%; the studies with unreported features and a moderate risk of bias had values of 50.0%, 37.5%, 37.5%, 25.0%, 50.0%, and 50.0%; and the studies at high risk of bias had values of 12.5%, 50.0%, 50.0%, 0%, 25.0%, and 25.0%.

**Figure 3:**
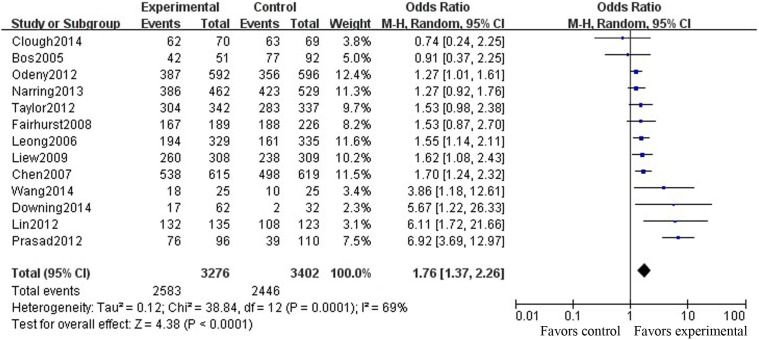
Comparison of the FUR between the SMS and the control groups.

**Figure 4:**
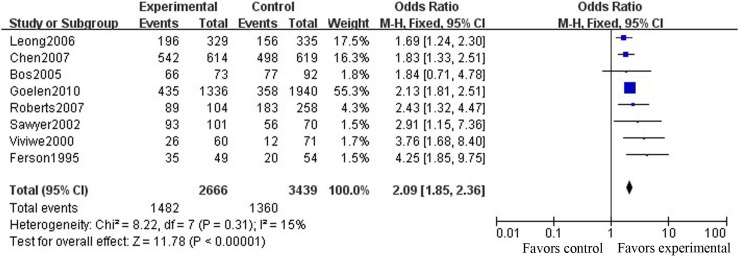
Comparison of the FUR between the telephone and the control groups.

The ORs of the included studies regarding the improvement of follow-up adherence in the SMS group compared with the control group (Figure 3) ranged from 0.74 to 6.92, and the pooled OR was 1.76 (95% CI [1.37, 2.26]; P<0.01). The ORs of the included studies regarding the improvement of follow-up adherence in the telephone group compared with the control group (Figure 4) ranged from 1.69 to 4.25, and the pooled OR was 2.09 (95% CI [1.85, 2.36]; P<0.01).

**Figure 5:**
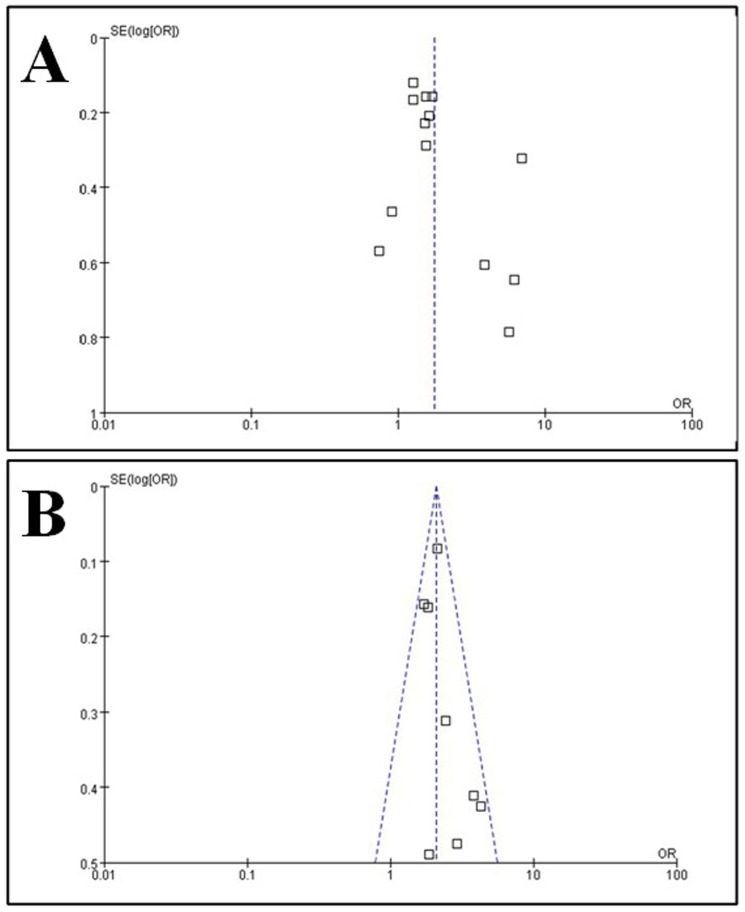
Funnel plots for publication bias testing. Panel A, SMS reminder effect; Panel B, telephone reminder effect. Each point represents a separate study on the indicated association. The vertical line represents the mean effect size. Generally, the points are distributed symmetrically as an inverted funnel, indicating minor publication bias.

To further assess the pooled results of the relationship between reminder effects and the FUR, Funnel plots (Figure 5) were applied for publication bias testing. We found that both SMS and telephone reminders were significantly related to improvement of the FUR, with no evidence of publications bias (Begg’s test, P = 0.161 (continuity corrected), Figure 5A; Begg’s test, P = 0.266 (continuity corrected), Figure 5B) and with high heterogeneity between studies (I^2^ = 69%, P = 0.001, Figure 3; I^2^ = 15%, P = 0.31, Figure 4). After omitting each study one by one and recalculating the combined estimates for the remaining studies, the main results were not notably altered (all P>0.05).

## Discussion

In the present study, we have provided a comprehensive review of the literature and quantitative estimates of associations between SMS/telephone reminders and the FUR from RCTs around the world. Our results indicated that SMS and telephone reminders both could significantly improve the FUR, and telephone reminders had a greater probability but also a higher risk of bias than did SMS reminders. At the time of our literature search, only 18 RCTs were identified in our systematic review. A total of 3276 patients with and 3402 patients without SMS reminders and 2666 patients with and 3439 patients without telephone reminders were described in these studies. There is RCT evidence of reasonable quality showing that SMS and telephone interventions aimed at improving FUR can be effective.

AFU is considered to play an essential role in chronic disease management characterized by long-term observation and is important to choose the optimal timing of surgery, deliver cascade of care, detect complications associated with the surgery, collect outcome data, and diagnose recurrent disease. [33] For example, a postoperative follow-up program is recommended for nearly all cancers in the National Comprehensive Cancer Network. [1] Patients with glaucoma also require life-long treatment and follow-up care to preserve vision. [5] Prolonged surveillance and medication can prevent deterioration from hepatitis B to cirrhosis or hepatocellular carcinoma. Loss to follow-up is the major reason for hepatitis recurrence. [6] The importance of follow-up is also emphasized for the management of coronary artery diseases, [8] cerebral infarction, [34] diabetes, [35] asthma, [36] chronic kidney disease, [7] obesity, [37] chronic sinusitis, [38] cataract, [39] and amblyopia, [40] among others. Practically, even a carefully designed treatment plan does not yield the expected results with a lack of adherence (patients’ behaviors in terms of taking medication, following diets, or executing lifestyle changes coinciding with healthcare providers’ health and medical advice). [41] In addition to the treatment effect, lack of AFU seriously affects clinical research implementation by undermining the internal and external validities of the findings [42], attrition bias [43], increasing the trial’s cost and duration and delaying the acquisition of important results [9].

An important aspect of health interventions in areas with limited resources is that they must be inexpensive and ideally take advantage of existing resources. [44] In the era of mobile information technology, mobile telephone communication has been suggested as a method to improve the delivery of health services around the world, and randomized trials of mobile health technology interventions have created a substantial evidence base for the management and prevention of a broad range of disorders. [12] In the present systematic review, we found that the pooled OR for the improvement of the FUR in the SMS group compared with the control group was 1.76 (95% CI [1.37, 2.26]; P<0.01) and the pooled OR for the improvement of the FUR in the telephone group compared with the control group was 2.09 (95% CI [1.85, 2.36]; P<0.01). Although telephone reminders had a greater probability than SMS reminders in improving the FUR, cell phone SMS interventions are believed to be more practical and well suited to different settings. After telephone numbers are collected, automated SMS reminders are presumably more efficient and less expensive than live telephone calls. [45] Furthermore, electronic mail reminders are mainly a focused mobile mode in addition to SMS and telephone reminders. Certain studies have demonstrated significant reductions in clinical non-attendance. [46] Considering the variability of usage, qualitative analysis was not performed in the present study [47].

Several limitations of this meta-analysis should be considered. First, a risk of bias existed in and varied between different studies. Considering adequate sequence generation, allocation concealment, blinding, the evaluation of incomplete outcome data, and lack of selective reporting, the majority of the studies on SMS reminders were at low risk of bias (evaluations >50%). However, for the studies on telephone reminders that were at low risk of bias, only the evaluation of incomplete outcome data accounted for more than 50%. The quality of allocation concealment and blinding was poorest for telephone reminders as more than 80% of the studies were at moderate or high risk of bias. Second, AFU in the included studies varied in its definition and calculation methods, although the measurements of AFU were interchangeable. Third, the specific nature of the interventions and their settings were not considered and discussed, including the ages and habits of the patients with mobile telephone usage, which might influence the effect of SMS and/or telephone reminders, [11], [48]. Fourth, studies that have used other terms, have included FUR as secondary endpoints, or utilized a different definition of FUR or different calculation methods may have been missed. One RCT that tested the efficacy of SMS reminders on adherence to antiretroviral therapy among patients attending a rural clinic in Kenya was not included, [49] because the primary outcome of this study was whether adherence exceeded 90% during each 12-week period of analysis and the 48-week study period but was not the actual proportion of patients attending their appointment (as defined in our current study). Therefore, selection bias may exist and the results of this study may not be actually applicable to all settings worldwide. Despite the above limitations, our meta-analysis found convincing evidence that SMS and telephone reminders both could significantly improve the FUR. Telephone reminders had a greater probability but a higher risk of bias than SMS reminders. Research on intervention strategies for improving patient adherence to follow-up is still limited, and more studies are required.

## Supporting Information

Checklist S1 **PRISMA checklist.**
(DOC)
